# Role-Based Framing of Older Adults Linked to Decreased Ageism Over 210 Years: Evidence From a 600-Million-Word Historical Corpus

**DOI:** 10.1093/geront/gnab108

**Published:** 2021-07-29

**Authors:** Reuben Ng, Nicole Indran

**Affiliations:** Lee Kuan Yew School of Public Policy, National University of Singapore, Singapore; Lloyd’s Register Foundation Institute for the Public Understanding of Risk, National University of Singapore, Singapore; Lee Kuan Yew School of Public Policy, National University of Singapore, Singapore

**Keywords:** Age stereotypes, Aging narratives, Historical analysis, Psychomics, Quantitative social science, Text as data

## Abstract

**Background and Objectives:**

Older adults are exhibiting greater diversity in their aging trajectories. This has led to movements by the World Health Organization and AARP to reframe aging. We compare role-based framing and age-based framing of older adults over 210 years—a time span beyond the reach of traditional methods—and elucidate their respective sentiments and narratives.

**Research Design and Methods:**

We combined the Corpus of Historical American English with the Corpus of Contemporary American English to create a 600-million-word data set—the largest historical corpus of American English with over 150,000 texts collected from newspapers, magazines, fiction, and nonfiction. We compiled the top descriptors of age-based terms (e.g., *senior citizen*) and role-based terms (e.g., *grandparent*) and rated them for stereotypic valence (negative to positive) over 21 decades.

**Results:**

Age-based framing evidenced a significantly higher increase in negativity (15%) compared to role-based framing (4%). We found a significant interaction effect between framing (age-based vs. role-based) and stereotypic content across 2 centuries (1800s and 1900s). The percentage of positive topics associated with role-based framing increased from 71% in the 1800s to 89% in the 1900s, with narratives of affection and wisdom becoming more prevalent. Conversely, the percentage of positive topics for age-based framing decreased from 82% to 38% over time, with narratives of burden, illness, and death growing more prevalent.

**Discussion and Implications:**

We argue for a more role-centric approach when framing aging such that age ceases to be the chief determinant in how older adults are viewed in society.

The word *old* is rooted in the Latin term *alere*, which means to nourish (Covey, 1988; Palmore, 2000). Yet, the meaning of the word is now so far divorced from what it originally meant. *Old* today carries negative connotations, especially when used to describe people. Aging has taken on the image of a debilitating condition—one marked by heightened dependence on others and a shift toward social and economic redundancy (Ng, Allore, et al., 2020; Thomas et al., 2014). However, such a skewed perception of aging no longer stands up to scrutiny. Older people now exhibit greater diversity in their aging trajectories, making age less relevant as a basis for evaluating individual competencies and needs (Neugarten & Neugarten, 1986). Following this, the field of gerontology has departed from a focus on the vulnerabilities associated with later life to the more positive aspects of aging (Pitt-Catsouphes et al., 2017). Consequently, there have been calls recently in both academia and public discourse to reframe existing notions of aging (Busso et al., 2019; Diehl et al., 2020; Jarrott et al., 2019; Ng et al., 2016; Ng, Lim, et al., 2020; Sweetland et al., 2017). Our study contributes to this discussion, specifically by exploring how role-based framing of older adults, relative to age-based framing, is linked to societal perceptions of older adults.

With older people forming an increasingly large subset of the population, questions about the appropriate nomenclature used to refer to them have surfaced (Barbato & Feezel, 1987; Nuessel, 1982, 1999; Palmore, 2000). The issue is highly subjective, where what appears relatively benign to one is pejorative to another (Barbato & Feezel, 1987). Words formerly believed to be acceptable—*elderly*, *senior*, *aged*, and even *older adults*—now border on the offensive. The reality is that in a youth-obsessed culture, any reference to old age is not a compliment (Burling, 2017). So long as that holds true, no word for people who are advancing in their years can remain unassailable (Barbato & Feezel, 1987; Burling, 2017). What this implies is a need to reframe perceptions of aging, a major part of which includes a discussion on the different terms used to refer to older people.

The use of age-based terms is closely intertwined with a process fundamental to human cognition: social categorization. Classifying individuals into social categories fulfills the adaptive need to bring coherence to the numerous and complex human attributes that make up the social world (Fiske & Neuberg, 1990; Liberman et al., 2017). Although functional in theory, scholars have argued that social categorization can be deeply problematic. Specifically, it may lead to a gross oversimplification of social realities, a homogenization of intragroup differences, and an accentuation of differences across groups (Gillespie et al., 2012). Individuals are then primed to form essentialist beliefs or stereotypes, which may in turn foster tensions among social groups (Bytheway, 2005). An insidious consequence of age-based categorization is that it obscures the heterogeneity of older adults in terms of cognitive and physical functioning. The implication of this is that old age becomes synonymous with ideas of frailty and mental deterioration (Ylänne-McEwen, 2000), which age-based terms such as *elderly* are thought to signify (Palmore, 2000).

Social role theory posits that beliefs about social groups are tethered firmly to the roles they are perceived to inhabit (Eagly, 1987; Eagly et al., 2000). Prior studies have highlighted that beliefs about older people tend to be multidimensional in nature (Brewer et al., 1981; Hummert, 1990; Schmidt & Boland, 1986). More precisely, subsumed under the superordinate category of older adults is a host of both positive and negative stereotypes (Brewer et al., 1981; Hummert, 1990; Schmidt & Boland, 1986). Examples of positive subtypes include the “John Wayne conservative,” the “liberal matriarch or patriarch,” and the “perfect grandparent,” while those of negative subtypes comprise the “severely impaired,” the “shrew/curmudgeon,” or the “inflexible senior citizen” (Hummert, 1990). It has been suggested that information regarding the roles of these subtypes supersedes age in predicting how older adults are evaluated (Kite et al., 2005).

An area of research that has recently piqued the interest of gerontologists concerns how perceptions of specific older adults differ from perceptions of older adults in general. Studies found that grandparents were described by respondents in personality-related terms which were mainly positive, while the broader category of older people elicited descriptors denoting mental and physical decline (Hoogland & Hoogland, 2018; Newsham et al., 2020). Consistent with social role theory, it is plausible that positive attitudes toward grandparents are largely informed by the keen awareness of their social roles (Hoogland & Hoogland, 2018). Hence, it would be worthwhile to consider how positive perceptions of grandparents can be integrated more broadly into perceptions of older adults as a group (Hoogland & Hoogland, 2018).

The significance of our study is twofold. Conceptually, this study is among the first to compare the narratives associated with age-based and role-based terms. While past studies have traced how age-specific terms used to refer to older adults have changed in meaning over time (Covey, 1988), research on role-based terms is still in its infancy, with observations from prior analyses restricted to small samples (Hoogland & Hoogland, 2018; Newsham et al., 2020). This study enhances existing research by elucidating how age-based and role-based narratives have percolated over the last 210 years, providing insight into the meanings ascribed to old age and the roles of older individuals throughout history. Next, it is important to acknowledge that older adults today are generally healthier than their counterparts in the past. Although typically perceived as a nonproductive group of retirees, potentially dependent on others, they often hold myriad roles—be it as grandparents, working professionals, or volunteers. Thus, from a practical aspect, our study points to a need to reframe existing notions of aging, particularly by promoting a greater emphasis on the roles undertaken by older people.

This study compares role-based framing with age-based framing of older adults over a period of 210 years. We set out to achieve two objectives and test two hypotheses. First, we compare how age-based terms and role-based terms differ in their association with societal perceptions of older adults. We hypothesize that role-based framing will be associated with more positive perceptions of older adults compared to age-based framing (Hypothesis 1). Second, to make sense of the differing implications of age-based and role-based framing, we compare the narratives associated with these terms. We hypothesize that role-based narratives have become more positive over time (Hypothesis 2).

## Materials and Methods

### Data Set

Although research on semantic change dates back to the previous century, the rising availability of historical data sets today has made this study more viable (Tsakalidis et al., 2019). Many social scientists have analyzed time-based corpora as a way of studying cultural shifts over time (Michel et al., 2011; Ng et al., 2015). Following a recent study by Ng and Chow (2020), our 600-million-word corpus—which spans 21 decades from 1810 to 2019—was created by integrating the Corpus of Historical American English (COHA) from 1810 to 2009 with the Corpus of Contemporary American English (COCA) from 2010 to 2019. The integration of both corpora formed the largest structured historical English corpus comprising over 150,000 texts collected from newspapers, magazines, fiction, and nonfiction. Grants from the National Endowment for the Humanities and the National Science Foundation enabled the creation of this extraordinary historical data set.

The COHA is a hundred times larger than other similar historical corpora and has been used to identify historical shifts in narratives in other influential studies (Ng et al., 2015). It includes over 100,000 sources including popular magazines, newspapers, fiction, and nonfiction. The subgenres are equally represented in the COHA from 1810 to 2009, rendering it well-balanced to capture “real-world” changes in both culture and society in the United States (Davies, 2012). It is also structured for frequency analysis of specific keywords as well as the context of the word used in each decade, hence allowing researchers to document and understand the dynamics of societal narratives.

The COCA is the largest balanced corpus of contemporary American English with over 1 billion words, including 20 million words every year from 1990. It is divided equally among spoken language, fiction, popular magazines, newspapers, and academic writing. The corpus is also updated every 6–9 months, making it valuable for studying linguistic changes in American English (Davies, 2009). According to cultivation theory (Gerbner et al., 2002), different types of media within corpora accurately reflect societal perceptions of various eras. The COCA is therefore a suitable platform for studying age-based and role-based narratives.

### Measurement of Age-Based and Role-Based Stereotyping

To measure age-based stereotypes over 210 years, all synonyms of “older adult” from 1810 to 2019 as listed in the Historical Thesaurus of the Oxford English Dictionary (Kay et al., 2009) and the Oxford Thesaurus (Waite, 2006) were compiled. A total of 18 synonyms emerged. Four of them (*emerit*, *gerontic*, *through-old*, and *old aged*) did not show up in the corpus; another three of them (*eldern*, *well-aged*, and *passing old*) appeared less than 10 times in 210 years and did not refer to an older adult. Eleven target synonyms were retained for analysis (e.g., *aged*, *elderly*, *old people*, *senior citizen*, *older adult*, *golden ager*). The top descriptors that co-occurred most frequently—also known as collocates—with each of these 11 synonyms were compiled per decade for 210 years based on the following inclusion criteria: (a) Lexical Proximity: collocate present within six words before or after the target word. Articles such as “the” and “a” were not included in the six-word lexical span. If the target noun was the first word of a sentence, collocates from the preceding sentence were excluded; (b) Relevant context: collocate referred to specifically an old person (checked by two raters); (c) Mutual Information Score of 3 and above, which indicates semantic bonding, meaning that the collocate has a stronger association with the respective synonym than other words in the corpus (Church & Hanks, 1990). This is an innovative application of concordance analysis known as “Psychomics,” replicated from previous studies to analyze societal stereotypes (Ng et al., 2021; Ng & Indran, 2021; Ng & Lim, 2020).

For role-based stereotypes of aging across 210 years, we replicated the aforementioned methodology to compile collocates related to familial roles of older adults. The terms were *grandparent*, *grandparents*, *grandfather*, *grandmother*, *grandpa*, *grandma*, *granddad*, and *granny*. Across the age-based and role-based terms, 21,978 collocates met our rigorous inclusion criteria. To test Hypothesis 1, each collocate was rated (by two raters) on a scale from 1 (*very negative*) to 5 (*very positive*). This has proven to be a valid and reliable method of measuring words associated with age stereotypes (Levy & Langer, 1994) and is congruous with past corpus-based studies (Ng et al., 2015; Ng & Chow, 2020). The methodology also builds upon literature on priming (Levy, 2009), which states that the repeated association of negative words with older adults—or words within close lexical proximity—increases implicit ageism. Very negative collocates were rated 1 (e.g., *abuse*, *molest*), neutral collocates were rated 3 (e.g., *wheel*, *journey*), and very positive collocates were rated 5 (e.g., *great*, *amazing*). The interrater reliability using Cronbach’s alpha was 0.972 (95% CI: 0.946, 0.986) for the scoring method. For every synonym per decade, we calculated a mean score which was then weighted (by the number of times the synonym appeared in that decade) to determine a Cumulative Aging Narrative Score for age-based and role-based terms, respectively, per decade.

### Analytic Strategy

Hypothesis 1 indicates that role-based terms of older adults are associated with less negative stereotyping compared to age-based terms. This was tested by analyzing the respective stereotypic trends over 21 decades (1810–2019) and by determining whether the respective slopes were significantly different. Hypothesis 2 states that role-based terms will be associated with more positive topics from the 1800s to 1900s, while age-based terms will be associated with fewer positive topics over the same period. Latent Dirichlet Allocation (LDA) is a natural language processing method used for topic modeling. By probabilistically assigning words into topics, LDA identifies latent topics and clusters of words that co-occur frequently (Blei et al., 2003). LDA was performed on the respective age-based and role-based collocates in the 1800s and 1900s. Thereafter, a two-way mixed analysis of variance (ANOVA) was conducted with age framing (age-based vs. role-based) as the between factor and stereotypic content of topic (positive/neutral vs. negative) across centuries (1800s and 1900s) as the within factor.

## Results

### Hypothesis 1: Role-Based Terms of Older Adults Are Associated With Less Negative Stereotyping Compared to Age-Based Terms Over 21 Decades

**Figure 1.**
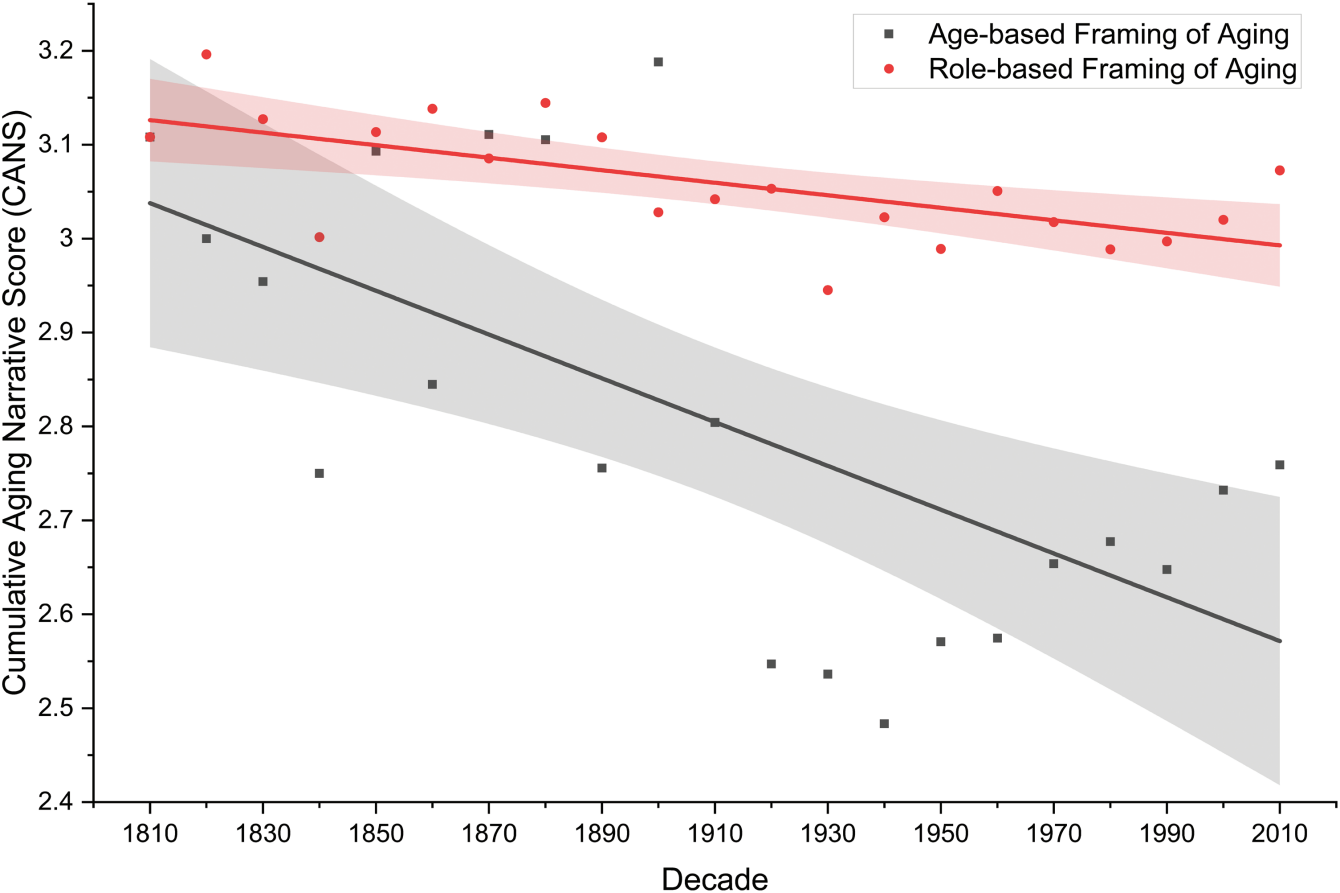
Cumulative Aging Narrative Score for age-based framing and role-based framing of older adults across 210 years from 1810 to 2019. *Note*: Age-based framing (through terms like “senior citizen”) is linked to a 15% decline in societal sentiments of older adults, compared to role-based framing (e.g., “grandparent”), which is linked to a 4% decline in societal sentiments. The respective shadings represent the 95% confidence band.

As hypothesized, role-based framing is associated with less negativity (β = −0.0067, *p* = .00146) compared to age-based framing (β = −0.023, *p* = .00148) over 210 years. The difference across both slopes reached statistical significance, *F* (1, 38) = 6.51, *p* = .0149, providing support for Hypothesis 1 (Figure 1). Specifically, for role-based framing, the intercept was 3.13 (*p* < .0001; 95% CI: 3.11, 3.15), indicating that role-based stereotypes of older adults were positive in 1810, with a 0.0067-unit decline per decade, resulting in a 4.28% decline over 21 decades. In contrast, the decline for age-based framing was more pronounced. Age-based stereotypes were positive in 1810 (intercept of 3.04, *p* < .0001, 95% CI: 2.97, 3.11), subsequently declining by 15.13% (0.023-unit per decade) over the 21 decades.

### Hypothesis 2: Role-Based Terms Are Associated With More Positive Topics From the 1800s to 1900s, While Age-Based Terms Are Associated With Fewer Positive Topics Over the Same Period

#### Topic modeling

Topics were generated for role-based and age-based terms across four genres (newspapers, magazines, fiction, and nonfiction) over two centuries (1800s and 1900s). Across the four genres, topics in the 1800s focused primarily on familial relations and politics, with positive themes related to wisdom and respect, as well as negative themes involving scandals and tension. In the 1900s, more positive narratives emerged, with emotive themes such as affection and sentimentality present across all genres, although negative themes of death also surfaced in newspapers and fiction. Meanwhile, topics for age-based terms evidenced a distinct shift from uplifting narratives of heroism and kinship in the 1800s to darker themes of illness, death, and burden in the 1900s across the same four genres. Fiction defied this trend by portraying older adults positively through romantic courtship and war heroism (Ng & Chow, 2020; see details in Table 1 and Supplementary Material).

**Table 1.** Comparison of Role-Based and Age-Based Narratives of Older Adults Across the Four Genres in the 1800s and 1900s

Genre	Role-based narratives 1800s	*Role-based narratives* *1900s*	Age-based narratives 1800s	*Age-based narratives* *1900s*
Newspapers	Death Political scandals	*Affection* *Wisdom* *Historical events* *Death*	Marriage Family life Courtroom proceedings	*Chronic conditions* *Social welfare* *Disability*
Magazines	Veneration War and history Familial relations Household affairs Celebration	*Familial relations* *Affection* *Learning* *Sentimentality* *War and history*	Family honor War heroism Love	*Death* *Caregiving* *Nursing homes*
Nonfiction	Royal matters Household affairs Knowledge Respect Intergenerational tension Danger	*Affection* *Caregiving* *Familial relations* *War and history*	Family relationships Marriage Royal families	*Death* *Illness* *Healthcare*
Fiction	Familial relations Wisdom Death Affection	*Sentimentality* *Affection* *Familial relations* *Household visits* *Death*	Courtship and romance War heroism Daily grind	*Showbiz* *Retirement homes* *Courtship and romance*

*Note:* The contrast is sharpest in the 1900s (italicized), where role-based framing attracted positive narratives of affection and wisdom, while age-based framing elicited negative narratives of disability, illness, and death.

#### Two-way mixed ANOVA

**Figure 2.**
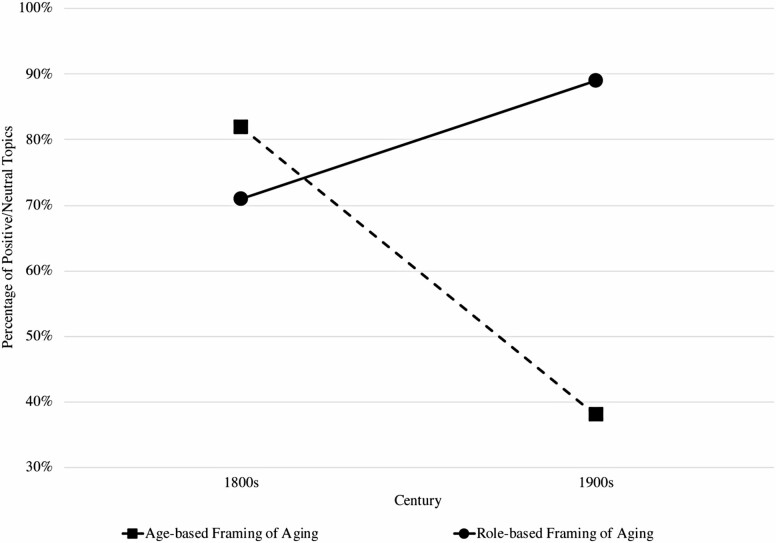
Percentage of positive/neutral topics for age-based versus role-based framing of aging over two centuries. *Note*: Results show a significant interaction effect between framing (age-based vs. role-based) and stereotypic content across centuries (1800s and 1900s). The percentage of positive/neutral topics associated with role-based framing increased from 71% in the 1800s to 89% in the 1900s with more positive narratives of affection and wisdom. In contrast, for age-based framing, the percentage of positive/neutral topics decreased sharply from 82% in the 1800s to 38% in the 1900s with more negative narratives of burden, illness, and death.

There was a significant interaction between framing (age-based vs. role-based) and stereotypic content across centuries (1800s and 1900s), *F* (1, 37) = 15.28, *p* < .0001, η*p*^2^ = 0.29. The main effect of framing also reached significance, *F* (1, 37) = 7.42, *p* = .01, η*p*^2^ = 0.17. The percentage of positive/neutral topics associated with role-based framing increased from 71% in the 1800s to 89% in the 1900s. In contrast, for age-based framing, the percentage of positive/neutral topics decreased sharply from 82% in the 1800s to 38% in the 1900s. Taken together, these results—visualized in Figure 2—provide support for Hypothesis 2.

## Discussion

Our study distilled the nuances of age-based and role-based narratives over the last 21 decades—a time span beyond the reach of traditional methods. We found that age-based framing is linked to negative societal perceptions of older adults. While the usage of age-based terms has practical value (Neugarten & Neugarten, 1986; Palmore et al., 2005), it may also have the paradoxical effect of reifying older adults as a distinct category. Age is cemented as a master status—a powerful social marker that effectively supplants all other attributes that make up one’s identity (Hughes, 1945; Palmore et al., 2005). The repeated usage of age-based framing is essentially a stark reminder to older adults that—for all their abilities, strengths, and contributions—their identities are defined first and foremost by their age. This may adversely affect how they perceive themselves, potentially rendering them susceptible to unfavorable health outcomes (Eibach et al., 2010; Kotter-Grühn & Hess, 2012; Ng et al., 2016; Ng, Lim, et al., 2020).

Previous studies have attributed the increasing negativity of age-based narratives over time to the rapid pace of aging and growing medicalization of later life (Ng et al., 2015; Ng & Chow, 2020). A point that must be acknowledged is that the “fourth age” or last stage of life brings with it a certain degree of vulnerability and dysfunctionality which science and policy are ill-equipped to solve (Baltes & Smith, 2003). However, being a heterogeneous population, attention has to be given to older people who are able to maintain optimal health even as focus is directed at those for whom aging can be profoundly challenging especially during the fourth age (Diehl et al., 2020).

Worth noting is that advances in science and medicine since the 1950s have resulted in a steady increase in average life expectancy and disability-free life expectancy (Crimmins, 2015; Crimmins et al., 2016). There is also strong empirical evidence that compared to those of the same age in earlier cohorts, older adults today fare better in cognitive performance and psychosocial functioning (Drewelies et al., 2019; Gerstorf et al., 2015, 2019, 2020; Hülür et al., 2019). The emergence of expressions such as “60 is the new 40” similarly alludes to the idea that relative to past generations, people today have reason to be cautiously optimistic about aging (Diehl et al., 2020). Yet, as indicated by our findings, negative views of aging and older adults have not only persisted but worsened, underscoring the pressing need to reframe aging in a way that considers both the gains and losses experienced in the aging process (Baltes & Smith, 2003).

Our finding that role-based framing of older adults is associated with less negative societal perceptions of older adults is a heartening one. It reveals unequivocally that when age is not foregrounded, negative stereotypes of older adults are less readily activated. There is evidence that implicit bias against older adults may be attenuated when messages about aging are reframed (Busso et al., 2019). To this end, a more role-centric approach to aging should be adopted such that age ceases to be the chief determinant in how older persons are viewed.

Nevertheless, it is important to explain why role-based framing of older adults is linked to a slight decline in societal sentiments over time. This could be due to a preoccupation with the idea that certain roles are technically being undertaken by older adults. Grandparents belong to a category of older persons, meaning role-based terms will inevitably acquire some of the same negative connotations conjured up by age-based terms (Caldas-Coulthard, 2020). In addition, role-based terms associated with grandparents have straddled the positive–negative continuum throughout history. According to Covey (1988), the expression “don’t teach your grandmother to suck eggs” was popularized in the 1800s, suggesting that grandmothers were commonly seen then as wise and experienced. In the late 1800s, the term *granny* was an insult targeted at older women. Today, although terms used to describe grandparents generally have positive connotations, *granny* and *grandmother* are sometimes used to symbolize antiquity, backwardness, or dullness (Caldas-Coulthard, 2020). Interestingly, the neologism *glamma*, a glamorous grandmother, has been gaining traction among grandmothers who feel that the term *grandmother* implies decrepitude (Zissu, 2011). On balance, though there is some negativity ascribed to role-based terms, specifically those related to grandparents, it pales in comparison with the negativity linked to age-based terms.

As highlighted by our thematic analysis, grandparents are valued for their expressions of affection—an invaluable emotional resource that promotes the social development of grandchildren (Mansson & Booth-Butterfield, 2011). Moreover, they play a vital role in childcare (Di Gessa et al., 2016; Shorey & Ng, 2020) and contribute immensely to society by allowing parents with young children to remain in the workforce (Goodfellow, 2003). Another key theme that surfaced in the role-based narratives is the idea that older adults are critical repositories of knowledge and wisdom. In the workplace, older adults are valuable assets constituting a major source of human capital. Their knowledge and expertise improve organizational performance and make them well poised to mentor younger staff (Vasconcelos, 2018). Aside from the familial and professional context, older adults are also key pillars of civil society. The overall rate of volunteerism among older people in the United States has gone up by 65% since 1974 (Gonzales et al., 2015). As a matter of fact, the contributions of volunteers among this cohort exceeded a staggering $19 billion in 2012 (Anderson et al., 2014).

Ageism is partly fueled by the notion that older adults do not contribute to society (Nelson, 2005). As such, concerted effort should be made to debunk misconceptions about older adults. While some of them are greatly disadvantaged and in need of both personal and social support, the majority are reasonably healthy with plenty to offer to society via both paid and unpaid roles (Riley & Riley, 1989). Previous studies have shown that views of older persons are positively predicted by the availability of information regarding their social roles (Hoogland & Hoogland, 2018). Hence, it will be useful to spotlight the roles played by older adults in society. Given that implicit associations are amenable to change (Busso et al., 2019), society will be nudged into viewing older adults more favorably, serving as a much-needed step toward curtailing ageism and its attendant costs. Older adults will also be encouraged to view later life as a period to embrace existing roles and assume new ones. This is particularly crucial because having positive beliefs about the aging process may improve self-evaluation and health (Ng et al., 2016; Ng, Lim, et al., 2020). Furthermore, in view of the discrepancy between the rising number of older people and the roles available to them (Riley & Riley, 1987; Riley, 1989), policymakers should consider creating adequate and suitable opportunities for older adults to ensure their strengths are well utilized.

This study had several limitations. First, the role-based terms were familial in nature and did not include broader professional and societal roles. Future studies could consider terms with cross-categorizations of age and profession such as ‘older scientist’ or ‘older doctor’. Though such terms are characterized by age-based framing—which we sought to avoid—insights into these terms could potentially elucidate whether older adults’ professional roles take precedence over their age. Second, we recognize that an emphasis on roles may perpetuate the view that older people are valuable only if they are productive—a concern frequently expressed by critical gerontologists (van Dyk, 2014). However, a focus on roles may help challenge the narrative of decline older people are often subject to as well as remind society of their untapped potential. Given that older adults are perceived more positively when their social roles are made salient as our findings show, future studies could explore other ways of framing older adults.

Third, this study only managed to compare data between two centuries. This broad-brush approach prevented us from understanding how the narratives were shaped by certain historical developments which led to shifts in attitudes toward older adults, for example, the advent of the printing press, the industrial revolution, and advances in geriatric care (Nelson, 2005). A promising avenue for future research would include disaggregation of the data by decade or year to draw conclusions at a more granular level and the triangulation of insights from multiple measurement techniques (Heidekrueger et al., 2017; Ng & Rayner, 2010; Sima et al., 2013). Future studies could also investigate language usage, frequency, and function of different parts of speech and the way these vary across genres of the historical corpus. Fourth, findings from this study may not generalize beyond the United States. Considering that age-related norms are likely to differ across cultural contexts, future studies could examine the nuances of age and role-based narratives in other cultures.

## Conclusions

As the population continues to age rapidly, the effects of ageism on both individuals and society will be felt more keenly. While an aging population brings about multiple challenges, it also presents innumerable opportunities that have yet to be leveraged. Given the frequent portrayal of old age as a period associated with many ills, we advocate for aging to be reframed in a manner that emphasizes the important roles of older adults and the tremendous value they add to society.

## Funding

This work was supported by the Social Science Research Council SSHR Fellowship (MOE2018-SSHR-004) and the Lloyd’s Register Foundation IPUR Grant (IPUR-FY2019-RES-03-NG). The funders had no role in study design, data collection, analysis, or writing.

## Conflict of Interest

None declared.

## Author Contributions

R. Ng designed the study, developed the methodology, analyzed the data, and wrote the paper. N. Indran co-wrote the paper.

## Data Availability

Data are publicly available at https://www.english-corpora.org.

## Supplementary Material

gnab108_suppl_Supplementary_Materials
