# Supplementary material for: Role-Based Framing of Older Adults Linked to Decreased Ageism Over 210 Years: Evidence From a 600-Million-Word Historical Corpus
Source: Gerontologist. 2021 Jul 29;62(4):589–97. doi: 10.1093/geront/gnab108 (PMC9019650; doi:10.1093/geront/gnab108)
Supplement: gnab108_suppl_Supplementary_Materials [file gnab108_suppl_supplementary_materials.docx]

**Supplementary Results for Hypothesis 2 (Topic Modelling)**

**Fiction 1800s.** Topic 1 is about ‘familial relations’ (maternal, son, wife). Topic 2 revolves around the ‘wisdom’ of grandparents (wise, venerable, respect) and Topic 3 around ‘death’ (die, death). Topic 4 focuses on grandparents’ ‘affection’ for grandchildren (care, smile, kiss).

**Fiction 1900s.** Words in Topic 1 evoke ideas of ‘sentimentality’ (remember, happy, picture). Topic 2 is about ‘affection’ (love, care, laugh, kid) and Topic 3 is about ‘familial relations’ (dad, wife, uncle). The remaining topics are related to ‘household affairs’ (visit, door, dinner) and ‘death’ (dead, bury, death).

Generally, fiction in both the 1800s and 1900s includes similar narratives, encompassing themes of affection and familial relations.

**Non-Fiction 1800s.** Topic 1 concerns ‘royal matters’ (royal, king, duke). Collocates in Topic 2 touch on ‘household affairs’ (master, ancestor) while those in Topic 3 refer to grandparents as repositories of ‘knowledge’ (historian, celebrate). Topic 4 makes reference to the etiquette of showing ‘respect’ (honor, respect, parents). Topic 5 is about ‘intergenerational tension’ (barbarity, ingratitude, fiendish, grandchildren). Words that imply ‘danger’ are present in Topic 6 (fear, rattlesnake).

**Non-Fiction 1900s.** Ideas of ‘affection’ are alluded to in Topic 1 (doting, care, love, gift). Topic 2 focuses on the ‘caregiving’ role undertaken by grandparents (caregiver, support, raise). Words in Topic 3 are about ‘familial relations’ (aunt, uncle, husband) and those in Topic 4 relate to ‘war and history’ (war, blood).

The only genre which consists of outrightly negative themes, particularly themes of danger and family conflict, is non-fiction, specifically in the 1800s. However, non-fiction took on a more positive tone in the 1900s, dwelling more on the social value of grandparents.

**Newspapers 1800s.** Topics centre predominantly on ‘death’ (deceased, death) and ‘political scandals’ (president, controversy, forge).

**Newspapers 1900s.** Topic 1 emphasizes grandparents’ expressions of ‘affection’ towards grandchildren (loving, care, doting). Collocates in Topic 2 hint at the ‘wisdom’ of grandparents (teach, learn). Topic 3 covers ‘historical events’ (war, serve, history, read) while Topic 4 is about ‘death’ (dead, murder).

Topics in the newspapers evolved from an emphasis on death and political controversies in the 1800s to that on grandparents as suppliers of affection, knowledge and wisdom in the 1900s—a marked shift towards positivity.

**Magazines 1800s.** Topic 1 is about the ‘veneration’ of the dead (remember, honor, respect). Topic 2 includes terms related to ‘war and history’ (war, colonel, return). Topic 3 contains ideas of ‘familial relations’ (maternal, grandson, cousin) and Topic 4 involves ‘household affairs’ (household, emigrate). Topic 5 has a ‘celebratory’ theme (marriage, religious, art).

**Magazines 1900s.** Topic 1 pertains to ‘familial relations’ (dad, husband, relative) and Topic 2 is related to ‘affection’ (love, care, gift). Topic 3 is about the value of ‘learning’ from grandparents (learn, lesson, kid). The remaining topics feature ideas of ‘sentimentality’ (photograph, youth) as well as ‘war and history’ (war, fight, letter).

Popular magazines moved from a general narrative of posthumous respect in the 1800s to a focus on the emotional and intellectual resources offered by grandparents in the 1900s.
